# Electrochemical Technology for the Detection of Tau Proteins as a Biomarker of Alzheimer’s Disease in Blood

**DOI:** 10.3390/bios15020085

**Published:** 2025-02-04

**Authors:** Jianman Wang, Xing Lu, Yao He

**Affiliations:** 1 Macao Translational Medicine Center, Macau University of Science and Technology, Taipa 999078, Macau SAR, China; 3240007730@student.must.edu.mo; 2 School of Pharmacy, Faculty of Medicine, Macau University of Science and Technology, Taipa 999078, Macau SAR, China; 3 Macao Institute of Materials Science and Engineering, Macau University of Science and Technology, Taipa 999078, Macau SAR, China; 4 Suzhou Key Laboratory of Nanotechnology and Biomedicine, Institute of Functional Nano and Soft Materials (FUNSOM), Soochow University, Suzhou 215123, China

**Keywords:** Alzheimer’s disease (AD), electrochemical detection, tau proteins, biosensors, point-of-care diagnostics

## Abstract

Alzheimer’s disease (AD) is a prevalent neurodegenerative disorder and a significant cause of dementia in elderly individuals, with a growing prevalence in our aging population. Extracellular amyloid-β peptides (Aβ), intracellular tau proteins, and their phosphorylated forms have gained prominence as critical biomarkers for early and precise diagnosis of AD, correlating with disease progression and response to therapy. The high costs and invasiveness of conventional diagnostic methods, such as positron emission tomography (PET) and magnetic resonance imaging (MRI), limit their suitability for large-scale or routine screening. However, electrochemical (EC) analysis methods have made significant progress in disease detection due to their high sensitivity, excellent specificity, portability, and cost-effectiveness. This article reviews the progress in EC biosensing technologies, focusing on the detection of tau protein biomarkers in the blood (a low-invasive, accessible diagnostic medium). The article then discusses various EC sensing platforms, including their fabrication processes, limit of detection (LOD), sensitivity, and clinical potential to show the role of these sensors as transformers changing the face of AD diagnostics.

## 1. Introduction

Alzheimer’s disease (AD) is the primary cause of dementia, which predominantly affects older adults, and its incidence is age-dependent [1,2,3,4]. It is important to note that AD is typically characterized by extracellular accumulation of amyloid β (Aβ) and intra-neuronal accumulation of neurofibrillary tangles (NFT)/tau tangles. The lack of effective treatments makes early and accurate diagnosis critical to intervene and mitigate the progression of AD [5]. Current treatments, such as N-methyl-d-aspartate (NMDA) receptor antagonists and acetylcholinesterase inhibitors, provide only temporary symptomatic relief in patients with advanced AD but do not cure them, thus stressing the importance of early diagnosis and timely intervention [4,6,7].

For the diagnosis of AD, the current effective technologies primarily rely on positron emission tomography (PET) and magnetic resonance imaging (MRI). While valuable, these technologies are expensive and accessible only to individuals with financial resources. Additionally, they exhibit low sensitivity in the early stages of AD [8,9,10]. Cerebrospinal fluid (CSF)-based detection of biomarkers such as phosphorylated tau (p-tau) and total tau (t-tau) is reliable but requires invasive lumbar punctures, making it impractical for large-scale or routine screening [11,12]. Blood-based biomarkers, particularly p-tau subtypes like p-tau181 and p-tau217, have emerged as promising low-invasive and cost-effective alternatives for early AD detection [13,14]. However, the low concentrations of tau in blood, approximately an order of magnitude lower than in CSF, present a significant analytical challenge [14,15,16].

Recent advancements in biosensing strategies have addressed some of these limitations [17,18,19,20,21,22,23,24,25]. Among various sensing approaches, electrochemical (EC) sensing technologies have demonstrated considerable promise due to their high sensitivity, rapid response times, affordability, and potential for miniaturization [26,27,28,29,30,31,32,33]. Unlike other methods, EC sensors are less affected by variations in temperature, pH, and usual interfering substances in biological samples, making them particularly suitable for clinical applications [34,35,36].

**Figure 1:**
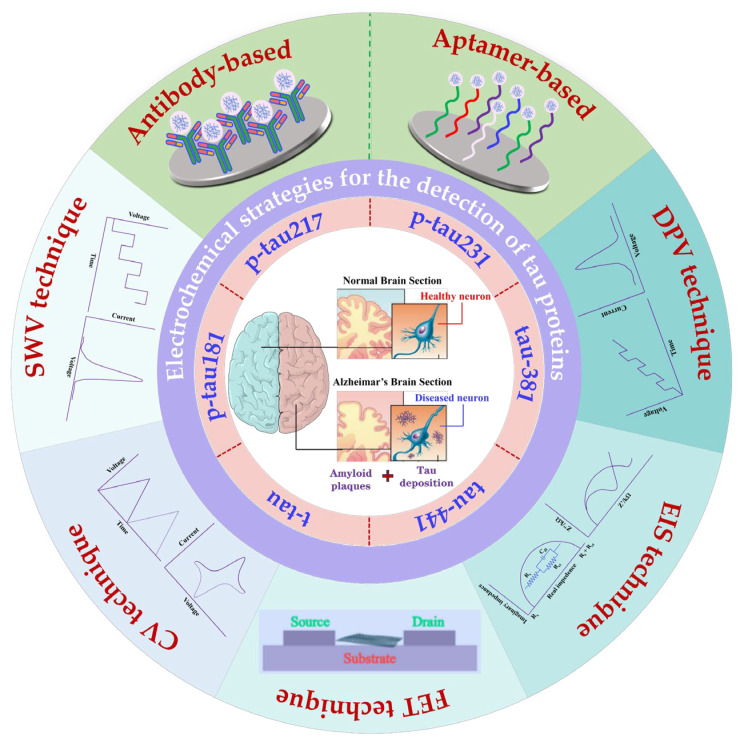
A schematic illustration of diverse EC strategies developed for detecting tau proteins associated with AD. EC, electrochemical; AD, Alzheimer’s disease.

As shown in Figure 1, this review highlights the latest developments in EC sensing technologies for the detection of AD-related tau proteins in the blood, including t-tau and p-tau isoforms (e.g., p-tau181, p-tau217, p-tau231, p-tau381, and p-tau441). We focus on key advancements in biosensors using antibody- and aptamer-based recognition elements and explore various EC methods, such as cyclic voltammetry (CV), differential pulse voltammetry (DPV), square wave voltammetry (SWV), electrochemical impedance spectroscopy (EIS), and field-effect transistor (FET) technologies. By detailing fabrication processes, analytical performance, and clinical applications, this review provides a comprehensive perspective on the transformative potential of EC biosensors in revolutionizing AD diagnostics.

## 2. AD-Associated Tau Proteins in Blood

Blood-based detection of tau proteins, particularly p-tau, represents a significant step forward in the development of accessible, non-invasive diagnostic tools for AD [37,38,39]. The occurrence of blood-based soluble p-tau proteins (p-tau181, p-tau217, and p-tau231) has greatly expanded the application of AD diagnosis and prognosis and clinical trial strategies [13,40]. The relationship between Aβ deposits and blood p-tau proteins may indicate the early onset of tau pathology or demonstrate initial physiological responses to Aβ accumulation that are not directly associated with tau tangle formation [39]. Due to limitations in detecting Aβ, such as small differences between plasma Aβ-positive and Aβ-negative individuals, lots of non-mass spectrometry (MS)-based assays have focused on plasma p-tau, specifically p-tau181, p-tau217, and p-tau231 [39,40]. Importantly, these biomarkers offer important information regarding AD progression and its value as a parameter for early detection and monitoring of AD.

Plasma p-tau181 is a widely recognized biomarker for AD, showing strong associations with amyloid PET imaging and CSF tau levels [41,42]. Its levels (healthy group: ~2.46 pg/mL) are elevated in the preclinical stages of AD and increase further with disease progression (mild AD patients: ~6.14 pg/mL) [41,42,43,44]. This biomarker effectively differentiates AD from cognitively normal individuals, mild cognitive impairment (MCI), and other neurodegenerative conditions [41,45,46]. Notably, higher baseline concentrations of plasma p-tau181 are associated with future AD pathology, even in individuals with normal cognition or MCI [41,43,45,47]. Plasma p-tau181 has shown predictive value for AD progression up to eight years before clinical diagnosis, and when combined with other biomarkers such as neurofilament light chain (NfL) and Aβ_42_, it improves predictive accuracy for clinical onset of AD [48].

Plasma p-tau217 has demonstrated superior potential compared to p-tau181 in tracking central nervous system (CNS) variations across the AD continuum. In specific scenarios, p-tau217 may provide better differentiation between AD and other neurodegenerative diseases. For example, a new electrochemiluminescence (ECL)-based plasma p-tau217 assay developed by Meso Scale Discovery (MSD) demonstrated superior discrimination between AD patients and controls compared to p-tau181 [49]. It is noteworthy that the fold change in plasma p-tau217 (3.9-fold) between AD (17.3 ± 7.4 pg/mL) and control group (4.4 ± 2.4 pg/mL) is consistently higher than in plasma p-tau181 (2.2 ± 0.7 vs. 1.3 ± 0.8 pg/mL, 1.7-fold) [49,50,51]. Research by Shorena Janelidze’s team has demonstrated that MS-based detection of plasma p-tau217 outperforms p-tau181 and p-tau231 in predicting Aβ status and tracking preclinical AD progression [52,53]. These studies indicate that plasma p-tau217 excels in clinical staging and may surpass plasma p-tau181 in distinguishing AD from normal aging and other neurological disorders [51,54,55].

Plasma p-tau231 has recently become a promising biological marker for monitoring the pathological state of early AD, with significant potential for use in clinical trials. Research by Nicholas J. Ashton et al. indicated that plasma p-tau231 levels rise earlier than p-tau181, coinciding with mild Aβ accumulation, before it becomes detectable by amyloid PET imaging [56]. Additionally, plasma p-tau231 reflects early tau deposition in the brain, further underscoring its potential for preclinical AD detection [55,57]. However, plasma p-tau231 has shown less effectiveness than p-tau181 in distinguishing AD from non-AD neurodegenerative conditions and has a lower detection rate for amyloid PET positivity compared to CSF p-tau231 [58]. These findings suggest that while plasma p-tau231 might reflect the earliest phase of AD pathology, other p-tau isoforms, such as p-tau181, could offer more reliable indicators of subsequent cognitive decline.

Beyond p-tau181, p-tau217, and p-tau231, other tau isoforms, such as tau381 and tau441, have garnered attention for their potential roles in AD diagnosis. Tau381, in particular, has been identified as a key p-tau protein for identifying AD in its earliest stages but is susceptible to interference from other neurodegenerative disease types [59,60,61]. Recent advancements, such as the development of aptamer–antibody sandwich biosensors, have enabled the detection of tau381 at concentrations as low as 0.42 pM (significantly lower than healthy population plasma tau concentration [~15.6 pg/mL]) [62], showcasing the potential for early AD detection [59].

Elevated blood levels of t-tau may reflect increased tau synthesis, but they are not always directly associated with neurodegeneration and are also elevated in other conditions associated with axonal damage, such as stroke and so on [63,64]. Recently, the development of monoclonal antibodies that specifically bind blood-based brain-derived tau (BBT) has effectively reduced the impact of tau from peripheral (non-brain) sources on detection [65]. Preliminary studies have shown that BBT has similar diagnostic performance to CSF t-tau proteins in distinguishing AD from controls or other neurodegenerative disorders [65,66]. However, their clinical utility hinges on their ability to consistently correlate with disease progression, cognitive decline, and other relevant clinical outcomes.

## 3. Application of EC Technologies in EC Biosensors

Tau protein detection has been achieved using many different EC biosensing technologies, including EIS, CV, DPV, and SWV. Such techniques allow for the detection of analytes with accuracy and quantification by monitoring critical EC parameters such as impedance, current, potential, or capacitance originating from biorecognition interactions [67]. Each method possesses unique performances that can be applied to different detection environments and analysis demands. For instance, a low amplitude alternating current (AC) signal is applied, and EIS is analyzed across multiple frequencies to evaluate the resistance to electron transfer at functionalized electrodes. Information obtained with this method is valuable about molecular interactions and interfacial dynamics [68]. In contrast, the CV technique is utilized to study reaction kinetics and the reversibility of a redox process so as to provide information on interfacial electron transfer mechanisms [69]. Additionally, DPV is well regarded for its ability to distinguish between analytes with unique peak potentials and has become a favorite method for the simultaneous detection of multiple targets [70].

Antibodies and aptamers are the most common biorecognition elements due to their specificity, versatility, and adaptability. The use of antibody- and aptamer-based EC biosensors for detecting tau proteins based on various EC technologies is explored in this section; their principles, recent advancements, and significance in early-stage AD diagnosis are discussed.

### 3.1. Application of EC Technologies in Antibody-Based EC Biosensors

EC biosensors based on antibodies exploit the strong specificity of the antigen–antibody interaction to allow sensitive target biomolecule detection. The antibody can capture antigens on the sensor’s surface or act as a receptor for the target molecules. The resulting detection is extremely low in concentration (e.g., picomolar to nanomolar) and very sensitive, which makes these interactions especially useful for AD diagnostics [71,72].

#### 3.1.1. CV Technology

CV is used as a tool for qualitative characterization of the EC reactions’ characteristics and behavior. It offers information on redox reaction thermodynamics, the kinetics of electron transfer at heterogeneous interfaces, and adsorptive processes or coupled chemical reactions [73]. More recently, CV has been effectively used to identify biomarkers correlated to AD, but the use of CV techniques specifically for targeting tau protein detection remains low.

To detect tau441 proteins in clinical samples for AD diagnosis, Karabiga et al. developed an immunosensor in 2020 [24]. This device integrates nanocomposites made of reduced graphene oxide and gold nanoparticles (rGO/AuNPs) onto a disposable indium tin oxide (ITO) electrode. The nanocomposite was functionalized with 11-MUA in order to improve the sensitivity, and the CV technique was used to track the construction progression of the sensor. The inter-peak potential difference (ΔEp) was observed to increase significantly, reaching 1.02 V after the formation of 11-MUA self-assembled monolayers (SAMs) on the rGO/AuNPs modified ITO electrode.

**Figure 2:**
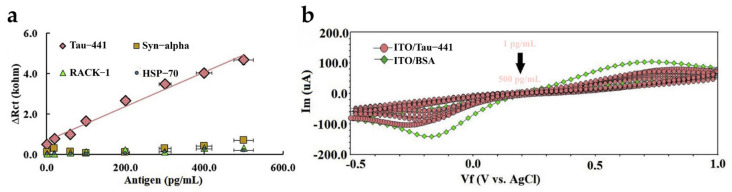
(**a**) Calibration plot showing the concentration-dependent response of the neuro-biosensor for tau441 detection using anti-tau under optimal working conditions; (**b**) voltammograms corresponding to varying concentrations of tau441. Copyright Elsevier (2020) [24].

As seen from Figure 2a,b, this device exhibited a strong linear relationship between a gradual decrease in peak current and tau441 concentrations (1–500 pg/mL). Data from an EIS response were consistent with the CV response. The sensor showed an excellent detection limit of 0.091 pg/mL, which demonstrated its ability to perform ultra-sensitive analysis of tau proteins.

Additionally, the biosensor exhibited high performance for tau441 detection in serum and CSF samples, with recovery falling in the range of 96 to 108%. The RSD of the slopes and intercepts of the used six biosensors were 3.02% and 3.41%, respectively, indicating that this novel platform possesses good reproducibility. Meanwhile, the impedance signal did not change significantly during the 8-week storage period, indicating the excellent stability of this system. The practicality and the cost-effectiveness of the practical clinical application were further enhanced by the use of disposable ITO electrodes. Overall, it is demonstrated that this rGO/AuNP-based neuro-biosensor holds the potential for selective and highly sensitive detection of tau441, a major biomarker of AD, and will have significant utility as a tool for early and specific AD diagnosis.

#### 3.1.2. SWV Technology

The high-amplitude differential pulse method, SWV, is a classical technique in differential pulse, characterized by superimposing a symmetrical square wave on a staircase potential to the working electrode [73]. This strategy is widely recognized for its excellent sensitivity, which is due to the fact that the net current is greater than either the reverse or forward component (as it is the difference between the two), and the sensitivity is higher than that of DPV (no reverse current is used) [73]. In recent months, SWV has attracted attention as an effective method for sensitive detection of tau proteins associated with AD.

**Figure 3:**
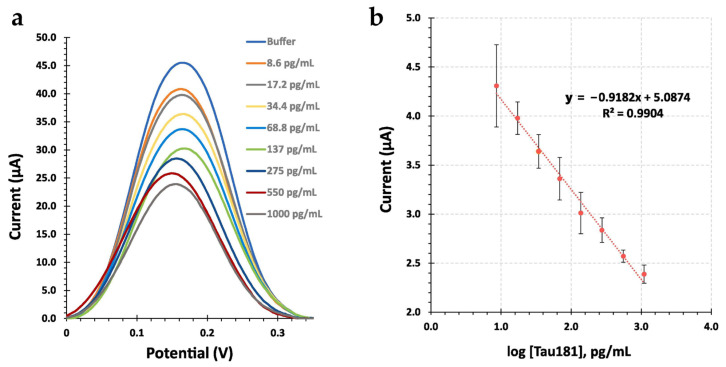
(**a**) SWV measurements and (**b**) calibration curve logarithm of the concentration of p-tau181 proteins against current. Copyright Elsevier (2022) [74]. SWV, square wave voltammetry.

In recent years, multi-walled carbon nanotube (MWCNT) has begun to be used for the detection of AD-related biomarkers due to its large surface area, good electrocatalytic properties, modification by other nanomaterials with stable physicochemical properties, and other advantages, thus enabling signal amplification as well as sensitivity enhancement [25]. For example, Schneider et al. (2022) came up with an advanced approach for preparing a novel EC immunosensor to detect p-tau proteins (p-tau181) [74]. The carbon screen-printed electrodes (C-SPEs) were modified with the MWCNTs and the platinum nanoparticles to develop a highly efficient platform for antibody immobilization in their research. The performance of the immunosensor is also assessed utilizing the SWV technique by changing the p-tau181 levels. The results in Figure 3a showed that the current response decreased with increasing p-tau181 concentrations. In Figure 3b, the calibration curve (current vs. log [p-tau181]) extended from 8.6 to 1100 pg/mL and had an LOD of 0.24 pg/mL, which is significantly lower than the plasma levels of p-tau181 in the healthy population (~2.46 pg/mL) and in mild AD patients (~6.14 pg/mL) [44]. The stability (recoveries greater than 87% in all three replicates of spiked serum samples analyzed) and high specificity (the effects of interfering substances [Uric acid, IgG, hemoglobin, and bovine serum albumin (BSA)] were < 8% when compared to the p-tau181-only solution) of p-tau181 detection in the complex serum environment demonstrated by this sensor shows its potential for clinical use.

Lastly, the SWV-based immunosensor provides a minimally invasive, highly sensitive, and easy-to-use methodology to quantify the levels of p-tau181 in clinical settings. In terms of operation and excellent analytical performance, it is a promising candidate for point-of-care diagnostic applications. In addition, it can enhance the existing clinical diagnostic techniques, representing another important aspect of AD research and the development of early AD diagnostic strategies.

#### 3.1.3. DPV Technology

Due to the extreme sensitivity, high resolution, and ability to separate analytes based on their different peak potentials, DPV has been widely used for detecting tau proteins. For example, in Li et al.’s work (2020), an EC immunosensor is designed to detect tau441 proteins using a gold electrode modified with a carbon nanocomposite film [25]. A nanocomposite using MWCNTs, reduced graphene oxide (rGO), and chitosan (CS) was able to improve the conductivity of the electrode while serving as a stable platform for the immobilization of antibodies. Gold nanoparticles (AuNPs) were functionalized with tau441 proteins for signal amplification. Measured by the DPV method, the sensor exhibited a dynamic range of 0.023 to 3.672 pg/mL with a very low detection limit (LOD) of 0.021 pg/mL, which is significantly lower than plasma tau concentration in the healthy population (~15.6 pg/mL) and AD patients (~53.9 pg/mL) [62]. In addition, as seen from their results, the anti-tau441 antibody had excellent specificity (the tau441′s peak current [41.44 ± 0.16 μA] was not significantly different from that of the mixed interfering substances [Human Serum Albumin (HSA): 39.7 ± 1.4 μA, α-Synuclein (α-Syn): 40.2 ± 1.5 μA, L-cysteine (L-cys): 40.4 ± 1.2 μA, Arachidonic Acid (AA): 39.46 ± 0.17 μA, Glutamic Acid (Glu): 39.93 ± 0.99 μA]), reproducibility (the relative standard deviation of the current density was 4.74% after testing of three electrodes) and stability (peak current retained 92.86% of its initial level during 11-day), with a dissociation constant (Kd) of 7.6 pM that showed a strong binding affinity between the antibody and antigen. Its potential for early AD diagnosis was validated using serum samples from healthy, MCI, and dementia patients.

A label-free EC immunosensor for detecting the novel biomarker of AD, cis p-tau, was introduced by Shiravandi et al. (2022) [75]. The gold electrode in the sensor was modified by a self-assembled monolayer (SAM) of 3 mercaptopropionic acid (MPA) and 11-mercantoundecanoic acid (MUA) to immobilize a monoclonal antibody directed toward cis p-tau. With the aid of the DPV technique, a strong linear correlation between current response and detectable levels of cis p-tau was observed over a range of 0.459 to 0.138 × 10^6^ pg/mL in both serum samples and phosphate-buffered saline (PBS), with correlation coefficients exceeding 0.99. In real serum samples, the detection performance of this sensor (healthy: 15.0 ± 0.8 pM, MCI: 59.0 ± 2.2 pM) was comparable to those of ELISA (healthy: 15.3 ± 1.2 pM, MCI: 60.3 ± 2.0 pM), indicating that it possesses good sensitivity. Additionally, the sensor possessed excellent stability (current declined by only 3.5% during the 90-day storage period) and was successfully validated with clinical samples, CSF, and serum of AD patients, demonstrating its prospect in diagnostic applications.

**Figure 4:**
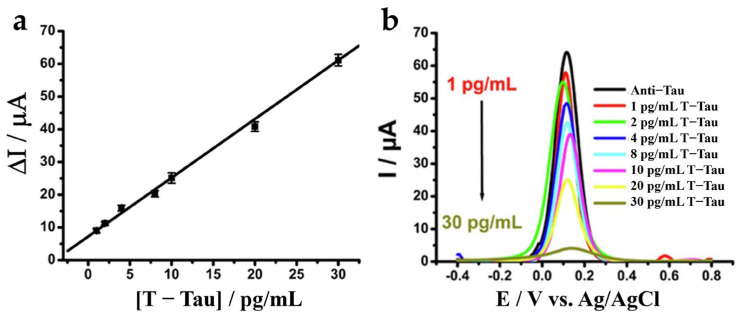
(**a**) Calibration curve of HBN-PDA/anti-t-tau immunosensor for t-tau concentration; (**b**) DPV curves for t-tau concentrations from 1 to 30 pg/mL. Copyright Elsevier (2023) [76]. DPV, differential pulse voltammetry.

In 2023, Simge Er Zeybekler continued to make further progress in the field by working on a novel hexagonal boron nitride (HBN)-based EC immunosensor that measures the levels of t-tau proteins [76]. To immobilize anti-t-tau antibodies without any additional crosslinking agents, this sensor employed a polydopamine (PDA)-coated HBN surface. An HBN-PDA nanocomposite-modified screen-printed carbon electrode (SPCE) was used as the surface to enhance antibody attachment and also provide a hydrophilic surface. As shown in the DPV results, this new sensor exhibited a linear detection range of 1 to 30 pg/mL and an LOD of 0.42 pg/mL, which was significantly lower than plasma t-tau levels in healthy populations (~3.07 pg/mL) (Figure 4a,b) [77]. Strong selectivity against common blood interferences was also demonstrated by the sensor, and a 96.62% recovery rate was achieved in artificial serum.

Recently, Li et al. (2023) created a printable vertical aligned graphene (VG) immunosensor that detected four AD-associated biomarkers simultaneously, with VG modified with gold nanoparticles (VG@nanoAu) [78]. Logarithmic linear relationships between levels of target proteins and peak current variations were recorded by the DPV method, with LODs of 0.072 pg/mL (Aβ_40_), 0.089 pg/mL (Aβ_42_), 0.071 pg/mL (t-tau), and 0.051 pg/mL (p-tau181). The sensor is made more useful by its integration with the smartphone interface. In addition, when a large number of interfering substances (Aβ_42_, t-tau, p-tau181, GLU, and HSA) were added, the ΔI did not change much and was much lower than the ΔI of Aβ_40_, indicating that this sensor possesses good specificity. Meanwhile, ΔI remains above 90% of the initial value over a 14-day storage period, demonstrating that it has excellent stability.

#### 3.1.4. Impedance-Based Technology

EC impedance-based sensors detect biomolecular interaction-mediated changes in charge transfer impedance (R_ct_) at the electrode surface as a label-free and high-sensitivity method for tau protein analysis.

In 2018, Carlin et al. created an EC biosensor that uses anti-tau antibodies immobilized to a gold (Au) electrode by N-hydroxysuccinimide ester coupling chemistry [79]. EIS was utilized to monitor the binding of Ab-Au to tau441 in the presence of the [Fe(CN)_6_]^3−/4−^ redox probe. The combination of the Ab-Au and tau441 resulted in a decrease in the charge transfer resistance R_ct_. After incubation with the interfering substance BSA (the most abundant protein in complex biological matrices, such as serum and CSF), all three Ab-Au surfaces showed tiny variations in impedance, indicating an excellent selectivity of the biosensor. Recorded by EIS, the sensor showed a linear response over μM to nM concentrations, and at these concentrations, negligible non-specific binding to BSA and no response to non-specific antibody surfaces like MOPC-21 was observed. It was, however, not validated in clinical samples and did not have enough sensitivity to meet diagnostic requirements.

In comparison, Kim et al. (2020) processed a much more advanced sensor array based on densely aligned carbon nanotubes (CNTs) to specifically detect four AD-related biomarkers in human plasma (Aβ_42_, Aβ_40_, p-tau181, and t-tau) [80]. High-density, unidirectionally arranged CNT sensor arrays reliably detect target analytes at concentrations as low as femtomolar due to the low density of inter-tube connections and the number of evenly distributed CNTs. The resistance variations in the array were recorded as a function of biomarker concentration, indicating a strong linear correlation between resistance variation and the logarithmic biomarker concentration over a range from 1 to 10^9^ pg/mL. R^2^ values above 0.99 were obtained from regression analysis with a coefficient of variation of resistance changes below 10%. The CNT-based sensor displayed much greater sensitivity than in Carlin et al.’s work (10^6^ pg/mL). They also achieved ultra-sensitive accuracy (2.13 fM for Aβ_42_, 2.20 fM for Aβ_40_, 0.112 pg/mL for t-tau, 0.215 pg/mL for p-tau181) and >93% recovery rate, effectively distinguishing AD patients from healthy controls. Among the EC sensors reported to date, this new platform achieves the lowest LOD for AD biomarkers and is capable of detecting four AD-related proteins simultaneously [80].

Impedance-based EC biosensors tailored for measuring tau protein levels in real blood samples have been focused on by recent developments. To provide an example, Hien T. Ngoc Le et al. (2021) presented a compact, low-price impedance-based EC biosensor for p-tau231 detection [81]. A design was developed with a forked finger electrode functionalized with a self-assembled monolayer to immobilize anti-p-tau231 antibodies. The changes in R_ct_ caused by p-tau231 binding to antibodies in human serum were recorded by EIS, obtaining a linear detection range from 10^−1^ to 10^4^ pg/mL and a detection limit of 140 pg/mL. This LOD is significantly lower than the typical p-tau231 concentration in CSF of patients with MCI (501 pg/mL) and AD patients (700 pg/mL), showing a strong prospect for early tau protein detection and clinical application.

**Figure 5:**
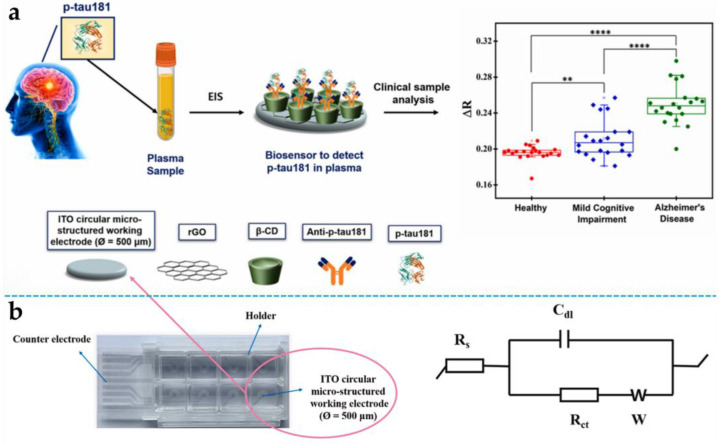
(**a**) Schematic representation of the impedance-based EC assay for plasma p-tau181 detection in clinical samples from AD and MCI patients as well as healthy controls and impedance detection of p-tau181 in human clinical plasma samples to identify patients with MCI (N = 20) and AD (N = 20), and healthy control individuals (N = 20) using the developed biosensor (****: *p* ≤ 0.0001, **: *p* ≤ 0.01); (**b**) images of ITO microelectrodes alongside Randle’s equivalent circuit model. Copyright Elsevier (2023) [82]. EC, electrochemical; AD, Alzheimer’s disease; MCI, mild cognitive impairment.

Based on this progress, Tieu et al. (2023) further enhanced the sensitivity for the detection of tau proteins in plasma by a novel developed impedance-based EC biosensor [82]. An indium tin oxide (ITO) microelectrode modified with a reduced graphene oxide/β-cyclodextrin (rGO/β-CD) nanocomposite and anti-p-tau181 antibodies was used in this system (Figure 5a). The advanced design achieved an exceptionally low detection limit of 0.92 fg/mL, significantly improving upon the 140 pg/mL limit reported by Hien T. Ngoc Le et al. Herein, the diameter of the Nyquist plot semicircle corresponds to R_ct_ in the Randles equivalent circuit model and is used as a parameter to evaluate the surface variation of the electrode (Figure 5b). The Randle equivalent circuit model was applied to fit the EIS–Nyquist plot, yielding an R_ct_ value of 110 kΩ for the bare ITO electrode. Subsequently, rGO/β-CD hybrid nanocomposites were electrochemically deposited on the ITO microelectrode, resulting in a significant decrease in R_ct_ to 54 kΩ. As a result, the sensor showed a linear response to p-tau181 concentrations from 10^−3^ to 10^3^ pg/mL and a Kd of 0.533 pM, reflecting strong binding affinity. Diagnostic sensitivity reached 95% and specificity reached 85% for distinguishing AD patients from healthy controls, while sensitivity and specificity for MCI patients reached 70%. Notably, this study was the first to quantify the Kd of the p-tau181 biomarker, offering a valuable tool for early, precise diagnosis of both AD and MCI.

**Figure 6:**
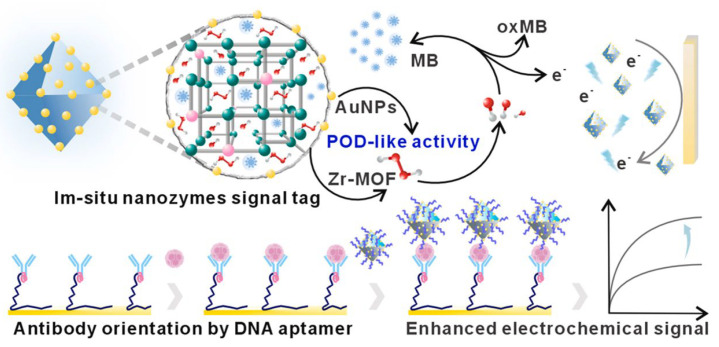
Schematic illustration of an EC immunosensor utilizing an in situ nanozyme signaling tag coupled with a universal antibody for the highly sensitive detection of AD-related biomarkers. Copyright Elsevier (2024) [83].

In addition, Chen et al. (2024) further advanced this technology with an EC immunosensor that can detect plasma tau proteins and BACE1 biomarkers [83]. As shown in Figure 6, this sensor illustrates a novel in situ enzyme catalytic amplification method coupled with DNA-mediated antibody orientation that provided an approach to boost sensitivity and selectivity. Core signal amplification was based on a zirconium (Zr)-based metal–organic framework (MOF) with peroxidase-like activity and encapsulating methylene blue (MB) as the electroactive molecule. On the enzyme-modified gold nanoparticles, hydrogen peroxide (H_2_O_2_) was used to produce hydroxyl radicals to achieve in situ catalysis, which was effective for signal amplification. While the primary function of the MOF is to facilitate the signal amplification, its specificity toward the analyte can be attributed to the immobilization of modified antibodies with anti-Fc DNA aptamers on the surface of Zr-MOF/MB/Au electrodes to specifically recognize tau and BACE1. Based on quantitative analysis of impedance, in a wide linear range of 0.01 to 10^6^ pg/mL, the immunosensor demonstrated LODs of 3.34 × 10^−3^ pg/mL for tau proteins, which is significantly lower than the plasma tau concentration in the healthy population (~15.6 pg/mL) [62], indicating that it can be sensitively used to detect attention deficit disorder biomarkers in complex biological samples.

In addition, Table 1 presents a comprehensive summary of recent progress in various EC immunosensors to detect AD-associated tau proteins, stressing the detection methods and analytical performance.

### 3.2. Application of EC Technology in Aptamer-Based EC Biosensors

Aptamers are short single-stranded DNA or RNA molecules that adopt a specific three-dimensional structure, which has been widely used in the development of EC sensors as a biological recognition element in recent years [84,85,86]. Compared with EC biosensors with antibodies as biological recognition elements, aptamers not only possess higher stability and lower production costs but also possess reusable regeneration capabilities [87]. Meanwhile, they are also synthesized in large amounts and with high purity and are easily functionalized by nanoparticles or enzymes to improve the performance of biosensors. These advantages make aptamers suitable for the preparation of fast, sensitive, and cost-effective EC biosensors.

**Figure 7:**
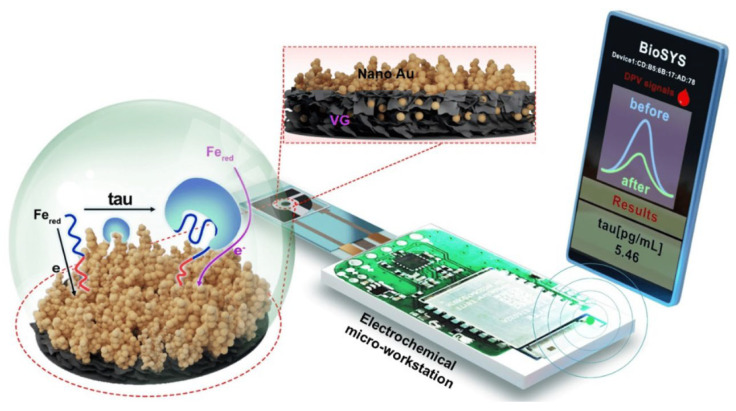
Schematic of the aptamer-based electrochemical biosensor for detection of serum p-tau231. Copyright MDPI (2022) [88].

Liu et al. 2022 reported a portable aptasensor platform for point-of-care testing (POCT) of tau proteins in blood [88]. As shown in Figure 7, a vertical graphene electrode modified with gold nanoparticles (VG@Au) was used as the working electrode. The large area and satisfactory electrical conductivity of the VG@Au improved the sensitivity of the sensor, achieving a lower LOD (0.1 pg/mL) than the tau concentration of healthy population (~15.6 pg/mL) and mild AD patients (~53.9 pg/mL) in plasma [62]. In addition, the linear detection range of the sensor was also extended to 1 ng/mL. Measurements by DPV showed that when tau proteins bind to the aptamer, the spatial structure of the DNA aptamer alters, impeding the electron transfer on the surface of the Au electrode, which results in reduced peak current. High specificity (the ΔI of tau protein > 12% while ΔI < 4% in Aβ, HSA, GLU, or AA) was shown by the sensor, with a Kd of 7.6 pM in human serum and stability of up to two weeks, ensuring robustness for early-stage AD diagnosis.

Cheng et al. (2023) presented a magneto-assisted enzymatic DNA walker system for the concomitant detection of amyloid beta oligomers (AβO) and tau proteins [89]. Herein, biotinylated aptamers immobilized on functional magnetic beads bind to the target (AβO and tau proteins) through competitive interactions, resulting in the release of the DNA walkers. Subsequently, the presence of double excision enzymes allows the free walkers to move across the electrode surface, releasing substrates corresponding to AβO and tau from the electrode surface, which enables one-to-more amplification of the signal. Current signals were found to be augmented by DNA assembly on a graphene electrode (GE), demonstrating detection ranges of 0–20 μg/mL for AβO and 0–10^6^ pg/mL for tau according to DPV measurements. An LOD of 1.28 pg/mL and 0.04 pg/mL for AβO and tau, respectively, was demonstrated by the biosensor, indicating its high sensitivity and multiplex detection fidelity.

In 2024, Kong et al. designed an EC biosensor to detect p-tau231 proteins using aptamers. To enhance electrical conductivity and signal amplification, gold nanoparticles were electrodeposited onto a glassy carbon electrode [90]. Specific interaction of p-tau231 with its aptamer was shown to form an aptamer–protein complex that impedes electron transfer, causing a proportional drop in current. Measurements by DPV technology, this novel sensor exhibited a linear detection from 10 to 10^7^ pg/mL and achieved a lower LOD (2.31 pg/mL) than the plasma p-tau231 concentration in the healthy population (~6.9 pg/mL) [91]. Furthermore, the sensor also possesses high repeatability (RSD of 3.27% for ten assays), stability (current attenuation of ~11.87% during a 7-day period), and reproducibility (8.08% RSD of the current response results in the six sensors) and can be an important tool for early tau protein detection.

## 4. Application of FET-Based Technologies in EC Sensors

FET-based biosensors are an innovative class of analytical tools that have garnered substantial interest due to their outstanding sensitivity and wide-ranging applications, capable of detecting analytes at extremely low concentrations, from pico- to atto-molar levels [92]. These sensors offer numerous benefits, including real-time monitoring, ultra-low detection thresholds, compact designs suitable for scalability, lower production costs, and compatibility with mass manufacturing processes [93]. These merits make it particularly suitable for clinical diagnosis, especially in the early diagnosis of AD.

**Figure 8:**
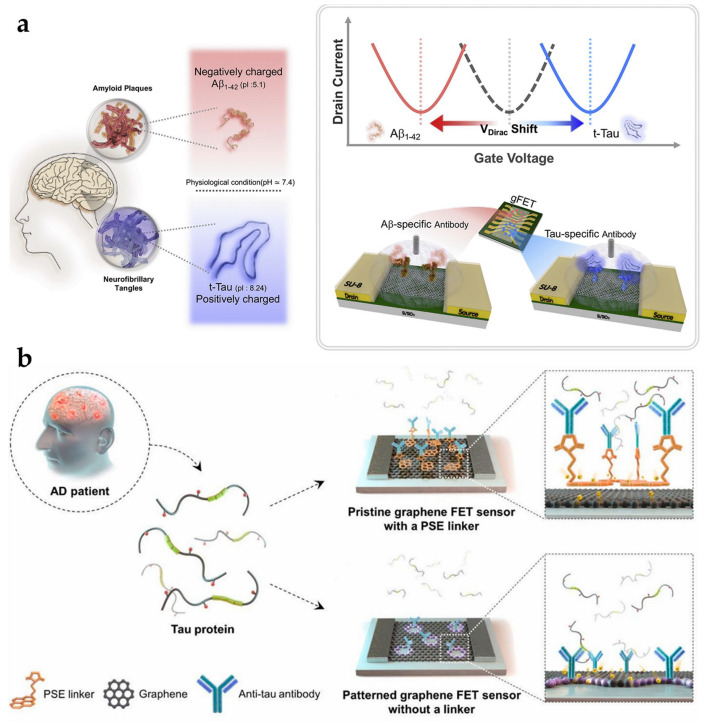
(**a**) Schematic representation of an electrolyte-gated gFET tau sensor for detecting AD-associated tau proteins. Copyright Elsevier (2021) [94]; (**b**) schematic of a gFET designed for the multiplexed detection of Aβ_1–42_ and t-tau biomarkers. Copyright Elsevier (2020) [92]. gFET, graphene-based field-effect transistor.

This includes, for example, Park et al. (2020), who developed a highly sensitive reduced graphene-based FET (gFET) biosensor for femtomolar detection of critical AD biomarker, Aβ_1-42,_ and t-tau in biofluids [92]. As shown in Figure 8a, this gFET exploits the distinct isoelectric points of the biomarkers to produce differential output signals. Physiologically, Aβ_1-42_, negatively charged, induces n-doping and causes a leftward Dirac point shift, while t-tau, which is positively charged, triggers p-doping, resulting in a rightward shift. Additionally, for targeted antigen identification, the rGO surface was modified with 1-pyrenebutyric acid N-hydroxysuccinimide ester (PBASE), followed by covalent binding of the antibody. The mechanism provides for a broad detection range (10^−1^ to 10^5^ pg/mL) and femtomolar sensitivity (significantly lower plasma t-tau levels than in healthy populations [3.07 pg/mL]) in media such as human plasma and artificial CSF [77]. The high specificity and sensitivity make the platform promising for early clinical AD diagnosis. This is also the first reported gFET that enables multiplexed detection of two AD-associated tau proteins with various electrical characteristics.

Furthermore, Kwon et al. (2021) introduced an advanced gFET immunosensor with an electrolyte-gated design that has a streamlined linker-free antibody immobilization strategy [94]. As shown in Figure 8b, when graphene is patterned and edge defects are created, the antibodies are immobilized directly on the oxidized edges of the graphene. The anti-tau antibody binds directly to edge defects in graphene without a linker and reacts with the tau protein, causing a change in the rate of change of the electric current by directly injecting electrons or holes into graphene. As a result, this novel platform achieves a low LODs of 0.01 pg/mL and is 2–3 times more sensitive than conventional graphene sensors with pyrene succinimidyl butyrate (PSE) linkers.

Compared to Park et al.’s reported linear range (10^−1^ to 10^5^ pg/mL), Kwon et al. extended detection from 10 fg/mL to 1 ng/mL, thus allowing the sensor to cover a wider concentration range [92,94]. Assays utilizing the plasma of AD patients showed that the current change rate was 3–4 times higher than that of the PSE-based gFET sensor. Further advancements in this technology highlight its potential for biomarker detection to assist in the early diagnosis of AD.

FET-based biosensors have recently been developed by researchers for the detection of other AD biomarkers, such as p-tau217. For example, Ciou et al. (2023) have produced a graphene solution-gated FET (SGFET) to detect p-tau217 with high sensitivity (LOD at 10 fg/mL was significantly lower than p-tau217 levels in plasma from healthy individuals [0.32~0.62 pg/mL]) [95,96]. The sensor in this work was based on a graphene oxide/graphene (GO/G) layered structure with the top GO layer functionalized with oxidative groups for covalent antibody attachment and the bottom graphene layer for signal transduction. By recording ΔV_CNP_ values that increase with increasing p-tau217 protein concentration, this device exhibited good specificity (the sensitivity in HSA solution remains at approximately 90% of the value measured in PBS), sensitivity (18.6 mV per decade), stability (<2% change in V_CNP_ value during 7-day storage period), as well as a linear electrical response to p-tau217 concentrations in the 10 fg/mL to 100 pg/mL rang. This research successfully detected p-tau217 using SGFET-based biosensors for the first time, providing a promising approach for early AD diagnosis.

Wang et al. (2024) further built on these innovations by developing a next-generation FET biosensor for the simultaneous detection of several AD biomarkers, p-tau217, Aβ_40_, Aβ_42_, p-tau181, and NfL [97]. The platform utilizes specific antibodies immobilized on the surface of the gFET, in which the antigen–antibody binding changes the surface charge density and changes the conductivity of the graphene channel. Additionally, the system also combines machine learning algorithms to combine biomarker panel results with clinical data to further improve the accuracy of diagnosis. This multi-biomarker approach obviously improved AD stage differentiation, realizing an area under the curve (AUC) over 0.94 in receiver operating characteristic (ROC) analyses. The closer the AUC value is to 1, the better the performance of the model, i.e., the greater the ability of the biomarker combination to discriminate between patients and non-patients. In comparison, this platform is 10 times more sensitive than commercially available ELISA assays and avoids the dependence on complex and expensive equipment for methods such as immunoprecipitation mass spectrometry, which has great potential for clinical applications.

In addition, Table 2 presents a summary of recent progress in EC sensing technologies to detect AD-associated tau proteins, stressing the detection methods and analytical performance.

## 5. Challenges and Future Perspective

Blood-based p-tau biomarkers, such as plasma p-tau231 and p-tau217, have demonstrated strong associations with AD pathology, from early Aβ accumulation to later-stage tau pathology [14]. While blood-based biomarker testing offers advantages like lower invasiveness, accessibility, and reduced costs compared to PET imaging or lumbar puncture [16], detecting these markers in blood remains challenging due to their extremely low concentrations and the difficulty of distinguishing pathological from normal states [50].

Recent advancements in EC sensing technologies have improved the reliable detection of blood p-tau. EC-based immunosensors convert biological interactions into electrical signals, enabling high sensitivity and cost-effective detection [100]. However, challenges such as the non-conductive nature of antibodies, reliance on nanomaterials for sensor fabrication, and susceptibility to environmental factors compromise reproducibility and stability. Furthermore, analyzing complex biological samples and performing real-time measurements remain significant hurdles, requiring innovations to enhance the practicality and robustness of these platforms [101].

Aptamer-based EC sensors provide a promising alternative to antibody platforms, offering high specificity, structural flexibility, and reduced production costs. However, practical challenges, including maintaining aptamer stability in complex environments, enhancing sensitivity, and reducing non-specific binding, must be addressed [102]. Future research should focus on integrating aptamers into scalable platforms while overcoming these obstacles.

Various EC techniques, including CV, DPV, SWV, and EIS, have been applied for tau detection, each with distinct strengths and limitations. Voltammetry techniques, particularly CV, DPV, and SWV, are widely used for their sensitivity, rapid response, and low detection limits. However, issues like false positives from physisorption and limitations in throughput hinder broader clinical adoption. Enhancing scalability and multi-sample capabilities is essential. EIS-based sensors offer high sensitivity for tau detection but require a deeper understanding of AC electrical circuits to improve charge transfer characterization. Multiplexed EIS sensors could be used to develop more comprehensive and earlier-stage AD diagnostics [103]. The FET-based sensor exhibits excellent sensitivity due to its ability to recognize biomarkers at femtomolar or picomolar levels. However, obstacles such as dependable signal modulation, along with the need for decreasing background noise and inconsistencies in equipment performance induced by material imperfections, remain critical challenges [104].

Most of the examples reviewed in the manuscript exhibited good sensitivity and reproducibility; however, challenges remain in detecting biomarkers at low plasma levels. To achieve more accurate and reliable detection, further improvements in sensitivity and reproducibility are needed. In the future, scientists should focus on developing systems that can detect multiple AD-related biomarkers and utilize advanced data analysis methods, such as machine learning, to promote accurate and early AD diagnosis. These technological advances may bridge the gap between experimental breakthroughs and clinical applications, providing practical additional options for the management of neurodegenerative disorders.

## 6. Conclusions

AD is one of the degenerative neurological disorders, and early detection is urgently needed to promote timely intervention and alleviate its development. The EC biosensor offers a reformatory diagnostic strategy for AD, which is characterized by its low cost, non-invasive design, and excellent sensitivity. The shift from CSF to blood-based biomarkers (such as tau, Aβ_40_, Aβ_42,_ and NfL) has significantly improved the accessibility of detection, making early diagnosis of AD more common.

Further enhancement of sensitivity and reproducibility is essential for detecting biomarkers at low plasma levels, which would improve the applicability of the biosensors in clinical diagnostics. Advances in nanotechnology, biometric systems, and data analysis are still critical to overcoming these puzzles. With the further development of technology, EC biosensors can revolutionize AD diagnosis by providing affordable, early, and widely available detection options, ultimately improving patient care and reshaping disorder management practices.

## Figures and Tables

**Table 1:** Comparison of the analytical performance of various antibody-based EC biosensors designed for the detection of tau proteins based on different EC technologies.

EC Techniques	Analytes	Real Sample	Linear Range	LOD	Ref.
CV	tau441	Human serum	1–500 pg/mL	0.091 pg/mL	[24]
SWV	p-tau181	Spiked serum	8.6–1100 pg/mL	0.24 pg/mL	[74]
DPV	tau441	Human serum	0.023–3.672 pg/mL	0.021 pg/mL	[25]
	cis p-tau	Human serum	0.459–0.138 × 10^6^ pg/mL	2.295 pg/mL	[75]
	t-tau	Artificial blood serum	1–30 pg/mL	0.42 pg/mL	[76]
	t-tau	Human serum	~pg/mL	0.071 pg/mL	[78]
	p-tau181			0.051 pg/mL	
EIS	tau441	PBS-T-BSA	10^8^–10^11^ pg/mL	10^6^ pg/mL	[79]
	p-tau231	Human serum	10^−1^–10^4^ pg/mL	140 pg/mL	[81]
Impedance-based	t-tau	Human plasma	1–10^9^ pg/mL	0.112 pg/mL	[80]
	p-tau181			0.215 pg/mL	
	p-tau181	Human plasma	10^−3^–10^3^ pg/mL	0.92 fg/mL	[82]
	tau	Human plasma	0.01–10^6^ pg/mL	3.34 × 10^−3^ pg/mL	[83]

EC, electrochemical; LOD, limit of detection; Ref., reference; PBS-T-BSA, phosphate-buffered saline (pH 7.2) containing 0.05% Tween-20 with 1% bovine serum albumin.

**Table 2:** Comparison of the analytical performance of various EC biosensors designed for the detection of tau proteins.

Type of EC Biosensor	EC Techniques	Analytes	Real Sample	Linear Range	LOD	Ref.
Aptamer-based	DPV	tau protein	Human serum	0.1–10^3^ pg/mL	0.034 pg/mL	[88]
	DPV	tau protein	Human serum	0–10^6^ pg/mL	0.04 pg/mL	[89]
	DPV	p-tau231	Human serum	10–10^7^ pg/mL	2.31 pg/mL	[90]
FET-based	FET	t-tau	Human plasma	10^−1^–10^5^ pg/mL	1 pg/mL	[92]
	FET	tau protein	Human plasma	0.01–10^3^ pg/mL	0.01 pg/mL	[94]
	FET	p-tau217	Human serum albumin	0.01–100 pg/mL	0.01 pg/mL	[95]
	FET	p-tau181	Human plasma	10^−6^–10^0^ pg/mL	>6.6 × 10^−4^ pg/mL	[97]
		p-tau217		10^−4^–10^3^ pg/mL		
Unlabeled	EIS	tau441	Bovine serum albumin	9.18–45.9 ng/mL	9.18 ng/mL	[98]
	DPV	tau	Human serum	0.1 pg/mL ~ 10 ng/mL	0.08 pg/mL	[99]

EC, electrochemical; LOD, limit of detection; Ref., reference; FET, field-effect transistor.
